# Proceedings of the Semi-Annual Meeting of the Esculapian Society, Held at Grand View, Ill., 25th and 26th May, 1859

**Published:** 1859-07

**Authors:** H. R. Payne, D. W. Stormont

**Affiliations:** President; Secretary


### PROCEEDINGS OF MEDICAL SOCIETIES.

### PROCEEDINGS OF THE SEMI-ANNUAL MEETING OF THE ESCULAPIAN SOCIETY, HELD AT GRAND VIEW, ILL., 25TH AND 26TH MAY, 1859.

The Society met in the Methodist Church, and was called to order by President, Dr. H. R. Payne. The minutes of the last meeting were read by the Secretary, and approved.

On calling the roll, twenty members answered to their names, or appeared during the meeting.

The Treasurer being absent, Dr. Gorham was appointed Treasurer *pro tem.*

The Censors recommended Drs. J. T. Pearman, of Elbridge, Ill., A. H. Kimbrough, of Georgetown, Ill., A. J. Miller, of Linton, Ind., and B. F. Keith, of Edwardsport, Ind., who were elected members of the Society.

On motion of Dr. Steele, adjourned, to meet at half past one P.M.

### AFTERNOON SESSION.

The Society met according to adjournment, the President in the chair.

Dr. Davis read a paper on a peculiar form of the blue disease of infants, which was discussed by several members.

The Secretary read a communication from Dr. Washburn, on the elements necessary for success in practice.

Dr. Steele moved, the thanks of the Society be tendered Dr. Washburn for his paper, and Secretary be instructed to notify him thereof. Carried.

Dr. Davis moved, that a committee be appointed to memorialize the County Board of each county in this district, to remunerate physicians for attending paupers not in the poor houses. Carried.

Drs. Davis, Chambers and Mitchell were appointed.

Dr. Chambers read on essay on uterine tumors, which gave rise to a general discussion.

Drs. Hogue, Smith and Spears were appointed a Business Committee.

On motion, adjourned, to meet at eight o'clock this evening.

EVENING SESSION.

The Society met as per adjournment, the President in the chair.

Dr. Davis delivered an excellent address to an attentive audience, on quackery, ancient and modern.

Dr. Chambers moved the thanks of the Society be tendered Dr. Davis, for his address, and a copy requested for publication. Carried.

The audience was dismissed.

Dr. York reported a case of injury of one eye, followed by serious disease of the other, which was discussed by several members.

Dr. York offered the following resolution:

*Resolved*, That this Society recommend to each of its members to make and report to every physician in his vicinity, the names of all such persons as shall willingly neglect or refuse to make proper remuneration for their services; and it shall be good ethics to refuse rendering their services to such persons.

After considerable discussion, on motion of Dr. Davis, the resolution was laid on the table until to-morrow.

Adjourned, to meet at eight o'clock A. M.

MAY 26, MORNING SESSION,

The Society met as per adjournment, the President in the chair.

The Business Committee submitted their report, which, after various amendments, was adopted, by the appointment of the following gentlemen to write on the subjects opposite their names, for the next meeting, viz.:

Dr. H. R. Payne—Effects of Malaria in Modifying Disease.

Dr. Davis—Diphtheritis.

Dr. Gorham—Delirinm Tremens.

Dr. F. R. Payne—Epilepsy.

Dr. McCord—Milk Sickness.

Dr. Spears—Ophthalmia.

Dr. Miller—Menorrhagia.

Dr. Ten Brook—Vomiting in Pregnancy.

Dr. Keith—Croup.

Dr. Hoge—Hemorrhoids.

Dr. Van Dyke—Pneumonia.

Dr. Chambers—Dropsical Effusions.
Dr. Pearman—Typhoid Fever.
Dr. Kimbrough—Neuralgia.
Dr. Hinkle—Chlorate Potass.
Dr. Swafford—The Hypophosphites.
Dr. Smith—Rheumatism.
Dr. Mitchell—Dysentery.
Dr. Frizell—Hooping Cough.
Dr. McAllister—Ergot in Hemorrhages.
Dr. Stormont—Veratrum Viride.

Dr. Steele reported a case of Hemoptysis cured by ergot. Dr. Swafford a case of Puerperal Convulsions. Both cases were extensively discussed.

Dr. Davis offered the following resolutions:

*Resolved*, That the President shall delegate to some member of this Society, the duty of drawing up a circular, setting forth the advantages of a combination of skill and experience as is attained in medical societies; and the imperative obligation resting upon all members of the regular profession, to unite themselves with some association of this kind; and that a copy of said circular be sent to each respectable practitioner within the bounds of this Society.

*Resolved*, That the Treasurer be instructed to pay into the hands of the selected member, funds to defray the expenses of the same.

The resolutions were adopted, and Dr. Davis was appointed to carry out their intention.

Dr. York's resolution, offered last evening, was called up. After some discussion, on motion of Dr. Steele, it was laid on the table until the next meeting.

On motion, adjourned to meet at half-past one o'clock.

AFTERNOON SESSION.

The President called the Society to order, as per adjournment.

Dr. Van Dyke reported a case of snake bite. Dr. Spears reported a case of Erysipelas. Both cases were discussed.

Kansas, Edgar County, was selected as the place for holding the next meeting; and Dr. Chambers was appointed to deliver the public address.

Drs. Hogue, Herrick and Spears were appointed a Committee of Arrangements.

Dr. Chambers offered the following:

*Resolved*, That the President and Business Committee be instructed hereafter to make no appointment of a member to write on a special subject, unless they are satisfied that such appointment will be filled. Adopted.

Dr. Chambers moved the thanks of the Society be tendered the Committee of Arrangements;—also, the Trustees of the Methodist Church, for the use of their house. Carried.

Dr. Stormont offered the following resolution, which was adopted.

*Resolved*, That the Secretary be instructed to notify all members of the Society, who have not attended the meetings for eighteen months from this date, that if they desire a continuance of membership, they are requested to be present, or furnish a paper, at the next meeting; or if they desire an honorable dismissal, they are required to give notice thereof to the President or Secretary; otherwise, they will be suspended.

The Secretary was ordered to furnish a copy of these proceedings to the *Chicago Medical Journal* for publication.

On motion, the Society adjourned, to meet at Kansas, on the last Wednesday in October next.

H. R. PAYNE, *President*.

D. W. STORMONT, *Secretary*.

